# Effect of Botulinum Toxin Injection and Extracorporeal Shock Wave Therapy on Nerve Regeneration in Rats with Experimentally Induced Sciatic Nerve Injury

**DOI:** 10.3390/toxins13120879

**Published:** 2021-12-09

**Authors:** Minsu Seo, Dongin Lim, Shengshu Kim, Taeyeon Kim, Bum Sun Kwon, Kiyeun Nam

**Affiliations:** Department of Physical Medicine & Rehabilitation, Dongguk University College of Medicine, Goyang 10326, Korea; msseo87@naver.com (M.S.); ydi06200@naver.com (D.L.); cocoa1668@naver.com (S.K.); tinaccjj@naver.com (T.K.); bskwon@dumc.or.kr (B.S.K.)

**Keywords:** botulinum toxin, shock wave, peripheral nerve injury, neural regeneration, functional recovery

## Abstract

This study was designed to compare the roles of botulinum neurotoxin A (BoNT/A) and extracorporeal shock wave therapy (ESWT) in promoting the functional recovery and regeneration of injured peripheral nerves. A total of 45 six-week-old rats with sciatic nerve injury were randomly divided into two experimental groups and one control group. The experimental groups received a single session of intranerve BoNT/A or ESWT immediately after a nerve-crushing injury. The control group was not exposed to any treatment. Differentiation of Schwann cells and axonal sprouting were observed through immunofluorescence staining, ELISA, real-time PCR, and Western blot at 3, 6, and 10 weeks post-nerve injury. For clinical assessment, serial sciatic functional index analysis and electrophysiological studies were performed. A higher expression of GFAP and S100β was detected in injured nerves treated with BoNT/A or ESWT. The levels of GAP43, ATF3, and NF200 associated with axonal regeneration in the experimental groups were also significantly higher than in the control group. The motor functional improvement occurred after 7 weeks of clinical observation following BoNT/A and ESWT. Compared with the control group, the amplitude of the compound muscle action potential in the experimental groups was significantly higher from 6 to 10 weeks. Collectively, these findings indicate that BoNT/A and ESWT similarly induced the activation of Schwann cells with the axonal regeneration of and functional improvement in the injured nerve.

## 1. Introduction

Peripheral nerve injury is a common cause of functional impairment, occurring in approximately 2.8–5.0% of North American and European trauma admissions due to various etiologies (e.g., fractures, laceration, and trauma) [1,2,3]. The regeneration of neural tissues is a great challenge in medicine due to their substantial impact on patients’ quality of life. The study of peripheral nerve injuries originated in the 19th century [4]. There are two main types of treatment for peripheral nerve injury: Surgical and non-surgical. Methods of surgical treatment include primary end-to-end repair and nerve grafts, and non-surgical treatments include physical therapy, electrical stimulation, hydrotherapy, and medications [5]. Attention has predominantly been focused on non-surgical therapeutic approaches, including new pharmacological or physical therapies to treat traumatic peripheral nerve injuries [6]. Two widely-used novel treatments, botulinum neurotoxin A (BoNT/A) and extracorporeal shock wave treatment (ESWT), have shown promise in modulating nerve regeneration after neural injury. The role of BoNT/A or ESWT in neural regeneration and functional recovery following peripheral nerve injury remains to be elucidated. The purpose of the present research is to evaluate and compare the roles of BoNT/A and ESWT in promoting functional recovery and regeneration associated with nerve injury in an experimental rat model. This is a preliminary study guiding novel therapeutics to improve neural regeneration in clinical scenarios.

The clinical use of BoNT/A is well established and is continuously expanding, especially in the treatment of peripheral nerve injury. BoNT/A is hypothesized to induce neural recovery by directly stimulating axonal regeneration in damaged peripheral nerves [7]. ESWT is a non-invasive treatment method used for musculoskeletal pain management using shock waves [8,9]. Recently, the application of ESWT has been expanded to non-musculoskeletal diseases, such as complex regional pain syndrome, ischemic heart disease, diabetic foot ulcers, and peripheral nerve injuries [10,11,12,13]. ESWT has been proven to upregulate angiogenesis and growth factors through the activation of endothelial nitric oxide synthase and vascular endothelial growth factor [14,15]. Most investigations of ESWT for peripheral nerve injury have focused on inflammation and neuropathic pain following neural damage [16]. However, the exact neural regenerative effects of ESWT remain unknown, and the definitive mechanism of ESWT on peripheral nerve damage is unclear.

The goal of this study was to investigate and compare neural recovery treated with BoNT/A or ESWT in rats with an experimentally-induced sciatic nerve injury. The experiments were performed by using the immunofluorescence staining, ELISA, real-time PCR, and Western blot. In addition, functional improvements were evaluated through an electrophysiological test and a gait functional evaluation.

## 2. Results

### 2.1. Effect of BoNT/A and ESWT on Schwann Cell Activity of Injured Sciatic Nerve (GFAP, S100β)

**Figure 1:**
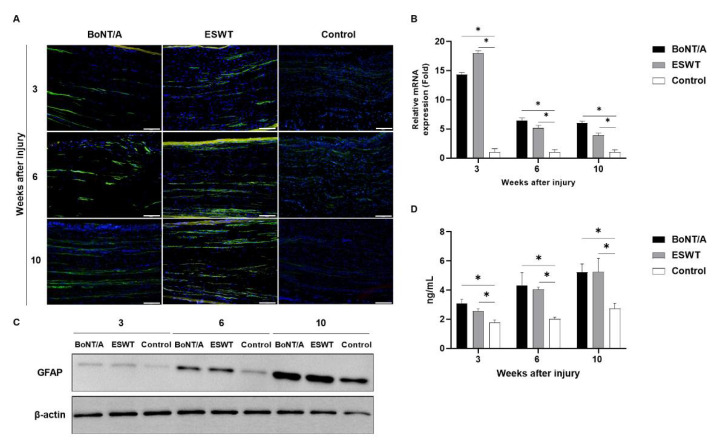
Effects of botulinum neurotoxin A (BoNT/A) and extracorporeal shock wave therapy (ESWT) on glial fibrillary acid protein (GFAP) expression of injured sciatic nerve: (**A**) Immunofluorescence staining image (scale bar: 100 μm; ×200). A higher GFAP expression was observed in the experimental groups. (**B**) RT-PCR revealed that GFAP expression was significantly increased in the experimental groups at 3, 6, and 10 weeks (* *p* < 0.05). Values are mean-fold changes in mRNA levels in the experimental groups relative to the control group. Error bars indicate standard deviation (SD). (**C**) Western blot indicated higher GFAP expression in the experimental groups. (**D**) ELISA. GFAP expression was significantly increased in the experimental groups at 3, 6, and 10 weeks (* *p* < 0.05). However, there was no difference between the experimental groups (*n* = 5 per group). Error bars indicate means ± standard error of the mean.

**Figure 2:**
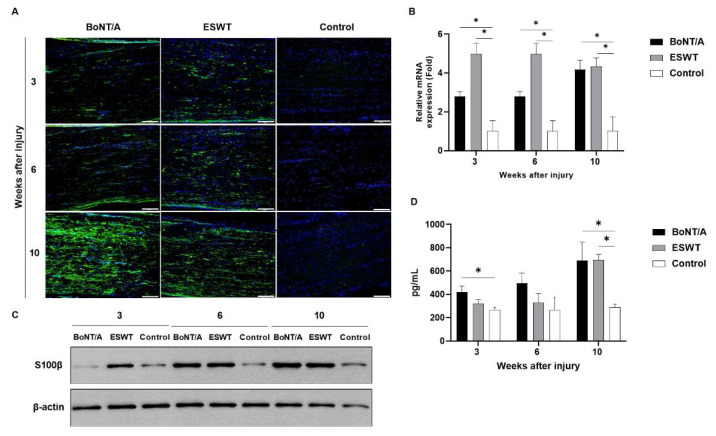
Effects of BoNT/A and ESWT on astroglial calcium-binding protein S100β (S100β) expression of injured sciatic nerve: (**A**) Immunofluorescence staining image (scale bar: 100 μm; ×200). A higher S100β expression was observed in the experimental groups. (**B**) RT-PCR revealed S100β expression was significantly increased in the experimental groups at 3, 6, and 10 weeks (* *p* < 0.05). Values are mean-fold changes in mRNA levels in the experimental groups relative to control group. Error bars indicate standard deviation (SD). (**C**) Western blot indicated higher S100β expression in the experimental groups. (**D**) ELISA. At 3 weeks, S100β expression was significantly increased in the BoNT/A group compared to control group (* *p* < 0.05). Compared to the control group, S100β expression was highly enhanced in the experimental groups at 6 weeks, but there was no statistical significance. With time, S100β expression was significantly higher in the experimental groups at 10 weeks (* *p* < 0.05). However, there was no statistically significant difference between the experimental groups (*n* = 5 per group). Error bars indicate means ± standard error of the mean.

A higher glial fibrillary acid protein (GFAP) and astroglial calcium-binding protein S100β (S100β) (green) expression in the experimental groups was observed in immunofluorescence staining (Figure 1A and Figure 2A). We used RT-PCR and Western blotting to detect the mRNA and protein expressions of GFAP and S100β in the injured nerves of rats from each group. The results demonstrated that, in comparison to the control group, mRNA and protein expressions of GFAP (Figure 1B,C) and S100β (Figure 2B,C) were remarkably increased in the experimental groups. ELISA also revealed a significantly higher GFAP expression in the injured nerves of rats in the experimental groups compared to that in the control group at weeks 3, 6, and 10, respectively (*p* < 0.05) (Figure 1D). S100β expression analyzed by ELISA was also significantly increased in experimental groups compared to the control group at week 10 (*p* < 0.05) (Figure 2D). At 6 weeks, S100β expression showed a higher trend in the experimental groups compared to the control group, but it was not statistically significant. There was no significant difference among the experimental groups.

### 2.2. Effect of BoNT/A and ESWT on Axon Regeneration of Injured Sciatic Nerve (GAP43, ATF3, NF200)

**Figure 3:**
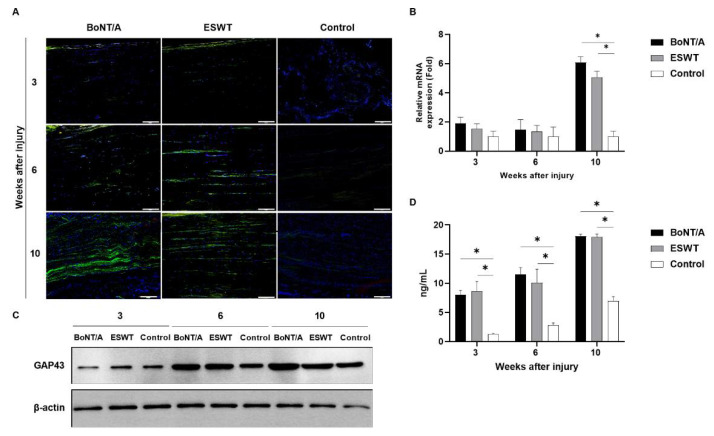
Effects of BoNT/A and ESWT on growth associated protein 43 (GAP43) expression of injured sciatic nerve: (**A**) Immunofluorescence staining image (scale bar: 100 μm; ×200). A higher GAP43 expression was observed in the experimental groups. (**B**) RT-PCR revealed that GAP43 expression was significantly increased in the experimental groups at 10 weeks (* *p* < 0.05). Values are mean-fold changes in mRNA levels in the experimental groups relative to control group. Error bars indicate standard deviation (SD). (**C**) Western blot indicated higher GAP43 expression in the experimental groups. (**D**) ELISA. GAP43 expression was significantly increased in the experimental groups at 3, 6, and 10 weeks (* *p* < 0.05). However, there was no difference between the experimental groups (*n* = 5 per group). Error bars indicate means ± standard error of the mean.

**Figure 4:**
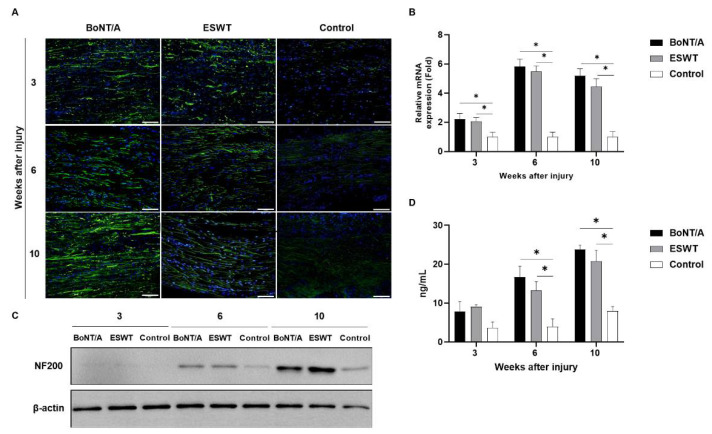
Effects of BoNT/A and ESWT on activating transcription factor 3 (ATF3) expression of injured sciatic nerve: (**A**) Immunofluorescence staining image (scale bar: 100 μm; ×200). A higher ATF3 expression was noted in the experimental groups. (**B**) RT-PCR revealed that GAP43 expression was significantly increased in the experimental groups at 6 and 10 weeks (* *p* < 0.05). Values are mean-fold changes in mRNA levels in the experimental groups relative to the control group. Error bars indicate standard deviation (SD). (**C**) Western blot indicated higher ATF3 expression in the experimental groups. (**D**) ELISA also showed higher ATF3 expression in the experimental groups at 3 and 6 weeks. ATF3 expression was significantly increased in the experimental groups at 10 weeks (* *p* < 0.05). However, there was no difference between the experimental groups (*n* = 5 per group). Error bars indicate means ± standard error of the mean.

**Figure 5:**
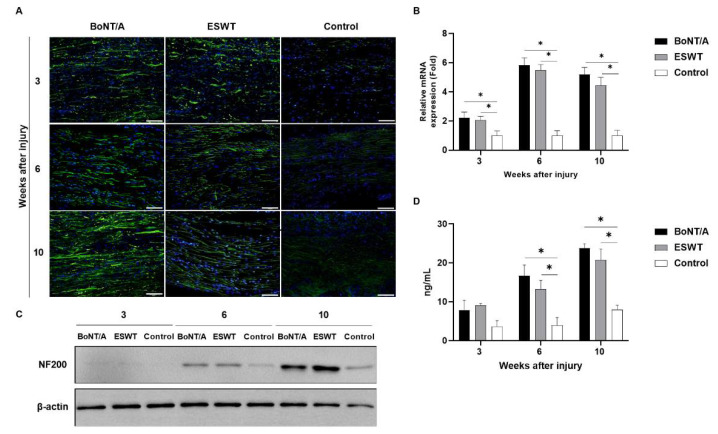
Effects of BoNT/A and ESWT on neurofilament 200 (NF200) expression of injured sciatic nerve: (**A**) Immunofluorescence staining image (scale bar: 100μm; ×200). A higher NF200 expression was observed in the experimental groups. (**B**) RT-PCR revealed that GAP43 expression was significantly increased in the experimental groups at 3, 6, and 10 weeks (* *p* < 0.05). Values are mean-fold changes in mRNA levels in the experimental groups relative to control group. Error bars indicate standard deviation (SD). (**C**) Western blot indicated higher NF200 expression in the experimental groups. (**D**) ELISA also showed higher ATF3 expression in the experimental groups at 3 weeks. With time, NF200 expression was significantly increased in the experimental groups at 6 and 10 weeks (* *p* < 0.05). However, there was no statistically significant difference between the experimental groups (*n* = 5 per group). Error bars indicate means ± standard error of the mean.

Immunofluorescence staining showed a profound increase in the expression of growth associated protein 43 (GAP43), activating transcription factor 3 (ATF3), and neurofilament 200 (NF200) in the experimental groups compared with the control group (Figure 3A, Figure 4A and Figure 5A). RT-PCR and Western blot also revealed higher expressions of GAP43 (Figure 3B,C), ATF3 (Figure 4B,C), and NF200 (Figure 5B,C) in the experimental groups compared to the control group. At 3, 6, and 10 weeks, in comparison to the control group, the level of GAP43 was significantly increased in the experimental groups (*p* < 0.05) (Figure 3D). ATF3 expression in the experimental groups was also significantly higher than that in the control group at week 10 (*p* < 0.05) (Figure 4D). At 6 and 10 weeks, the experimental groups demonstrated significantly higher levels of NF200 than those for the control group (*p* < 0.05) (Figure 5D). There was no statistically significant difference among the experimental groups.

### 2.3. Effects of BoNT/A and ESWT on Functional Recovery after Sciatic Nerve Injury

#### 2.3.1. Compound Muscle Action Potential (CMAP) Amplitude

**Figure 6:**
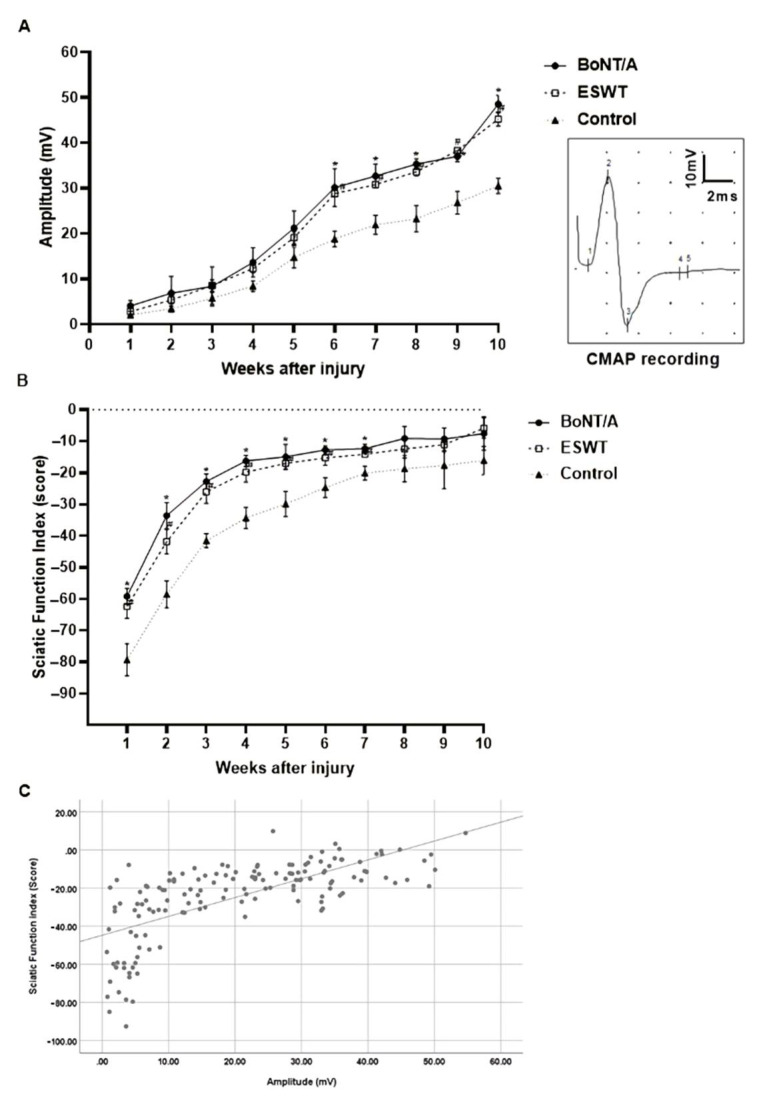
Effects of BoNT/A and ESWT on functional recovery after sciatic nerve injury: (**A**) CMAP amplitude (mV), representative recording of CMAP and (**B**) SFI (score) were measured weekly for up to 10 weeks following neural damage (*n* = 5 per group). (**A**) The experimental groups showed a statistically significant increase in amplitude from week 6 onward (* *p* < 0.05 for BoNT/A vs. control; # *p* < 0.05 for ESWT vs. control). (**B**) From weeks 1 to 7, in comparison with the control group, both BoNT/A and ESWT groups observed a statistically significant increase in SFI (* *p* < 0.05 for BoNT/A vs. control; # *p* < 0.05 for ESWT vs. control). However, there was no difference between the three groups after 8 weeks. (**C**) Correlation between the SFI (score) and CMAP amplitude (mV). There were significant correlations between SFI score and CMAP amplitude (r = 0.786, *p* < 0.05). Data presents as means ± standard error of the mean.

We measured the CMAP amplitude to assess the effects of BoNT/A and ESWT on nerve functional recovery following nerve injury by electrophysiological tests [17]. The results demonstrated a gradual increase in amplitude in both experimental and control groups, indicating a spontaneous recovery of motor function. After 6 weeks, in comparison with the control group, both BoNT/A and ESWT groups observed a statistically significant increase in amplitude (*p* < 0.05) (Figure 6A). However, there was no significant difference in amplitude between the BoNT/A and ESWT groups.

#### 2.3.2. Sciatic Function Index

The sciatic functional index (SFI) is a metric that is widely used by researchers who study the pathology and potential treatment of nerve injury. SFI was also used to assess the effects of BoNT/A and ESWT on the recovery of motor function in rats with neural damage. The results showed a gradual increase in SFI in the control group, indicating a spontaneous recovery of motor function. During the 10-week period, the SFI in the experimental groups also gradually increased, and the SFI in BoNT/A and ESWT groups was significantly higher than that of the control group from 1 to 7 weeks (*p* < 0.05) (Figure 6B). However, after 8 weeks, there was no significant difference among the three groups. Using Pearson’s correlation coefficient, we evaluated the correlation between the SFI (score) and CMAP amplitude (mV), which obtained a significant result (*r* = 0.786, *p* < 0.05) (Figure 6C).

## 3. Discussion

This is the first study to directly compare BoNT/A and ESWT in the context of neural recovery. The overall purpose of this investigation was to elucidate future therapeutic approaches to promote nerve regeneration in various clinical settings. This study demonstrated that BoNT/A and ESWT treatments were equally effective in nerve regeneration and functional recovery after peripheral nerve injury in rats. The electrophysiologic, clinical behavioral data, and neural tissue analysis presented here suggest that improved functional recovery was achieved via the activation of Schwann cells and axonal regrowth following BoNT/A or ESWT.

In a recent study, it was reported that BoNT/A not only reduced thermal and mechanical hyperesthesia in the damaged paws of rats subjected to chronic constriction injury, but also promoted the functional recovery estimated by the analysis of footprint walking tracks [18,19,20,21]. It is useful to reiterate that, following sciatic nerve damage combined with Wallerian degeneration, Schwann cells play a principal role [22]. The interaction between Schwann cells in the damaged nerve is crucial to induce a beneficial environment for maturation, sprouting, and regrowth of axons [23]. It is known that, following sciatic nerve damage, Schwann cells begin to proliferate and progressively enhance the expression of GFAPs and S100β, proteins expressed by nonmyelinating and myelinating Schwann cells, respectively [24,25,26]. It was confirmed that our results also suggested a direct interaction between BoNT/A and Schwann cells, similar to the previously reported data in vitro studies [27]. A recent study demonstrated that a single intranerve injection of BoNT/A increased the number of regenerated myelin fibers and the speed of axonal elongation following the complete crushing injury of peripheral nerves [7]. Regenerating axons revealed a concurrent expression of ATF3, GAP43, and NF200, with profound expression in BoNT/A and ESWT groups compared with the control group in our study. Based on previous investigations proving high efficacy at low concentrations with little side effect, a single session of intranerve BoNT type A (7 U/Kg; 336 pgtox/kg body weight) was used in this study [18,28]. However, there is no consensus as to the proper number of sessions and the method of BoNT/A injection (e.g., intranerve, intramuscular, intradermal, intrathecal, etc.) for peripheral nerve regeneration.

Extracorporeal shock waves at lower energy improve regeneration in several tissues, such as wounds, pressure ulcers, and chronic tendinopathy [29,30,31,32]. Several recent studies have investigated the role of shock waves in regeneration after peripheral nerve damage. Based on the propagation pattern of its wave, ESWT can be classified into two main modalities: Focused and radial ESWT [33]. Focused ESWT is generated by the probe, and the shock waves converge to the treatment site, whereas radial ESWT has maximum energy at the probe tip, and the waves are distributed radially into the tissue. Both focused [20,34,35,36] and radial [37] ESWT are equally effective in promoting nerve regeneration; however, focused ESWT yielded higher benefits in one study [38]. In our study, focused ESWT was used. There is no consensus as to the proper number of sessions and impulses of shock waves. However, three to four hundred shock waves, with a frequency ranging between 3 and 4 Hz in single or multiple daily sessions, are usually recommended [20,35,39]. One study showed that rats received 900 impulses of ESWT observed interrupted axons, while ESWT of 1500 impulses induced complete axonal degeneration [37]. These findings suggest that multiple impulses damage nerve tissue. The low energy flux density (EFD, in mJ/mm^2^) of ESWT reported in previous studies was 0.09–0.1 mJ/mm^2^ [34]. In our study, 400 impulses and 0.098 mJ/mm^2^ of low EFD were used. A further study is needed to determine the type of ESWT (focused or unfocused) and application parameters (number of sessions and impulse repetitions, frequency of strikes, and shock wave intensity) for better neural recovery. A single exposure to ESWT following sciatic nerve reconstruction via reversed autograft led to significantly improved voluntary motor function compared to the control at weeks 4 to 10 [37]. This finding suggests that the effect of ESWT is sustained up to 10 weeks, which is consistent with our findings; this is also similar to the duration of BoNT/A effects. The underlying mechanisms include enhanced angiogenesis and growth factor synthesis, as well as modulation of the inflammatory response [36,39,40]. In a previous study, ESWT showed an advantageous effect on Schwann cell isolation and culture, which correlates with our results [41]. Furthermore, increased axonal sprouting was also shown via a higher expression of GAP43, ATF3, and NF200 after ESWT, consistent with our results. These results support the experimental evidence that physical modalities such as ESWT could enhance an alteration in the microenvironment in the neural regenerative process.

The experimental groups, which were treated with BoNT/A or ESWT, showed significantly higher CMAP amplitude levels at 6–10 weeks compared with the control group, which only sustained a nerve injury without any treatment. The tibial nerve CMAP amplitude was normal and approximately 30 mV in 6-week-old rats before the nerve crush injury. A remarkable decrease in amplitude occurred after the nerve crush injury, indicating a successful induction of axonotmesis with Wallerian degeneration [42]. The sciatic nerve amplitude recovered similarly in both BoNT/A and ESWT groups from week 6 to 10 compared to the control. The amplitude at week 10 in both experimental groups was measured at approximately 50 mV, which was equal to the undamaged side. This electrophysiologic finding revealed a 1.66-fold axonal regeneration compared with the 30-mV amplitude in the control group at week 10. Additionally, the SFI established that the gait pattern was significantly improved in the experimental group from weeks 1 to 7 compared with the control group. This trend persisted until week 10, suggesting that BoNT/A or ESWT contributes to faster functional recovery. These results are consistent with previous experimental and clinical observations [36,39,43]. The difference between the experimental groups and the control group was reduced after 7 weeks due to the ceiling effect of SFI. While the SFI shows sudden recovery during specific periods, CMAP amplitude tends to recover in a relatively linear manner with time. As in previous studies in which the SFI shows rapid recovery values at the early phase after injury in the sciatic nerve crush injury model, reaching a plateau thereafter, our study also showed a similar pattern [44]. This suggests that the SFI showed a ceiling effect in the earlier stage as the degree of the nerve crush injury in our study was not a neurotmesis but a partial axonotemsis state. Therefore, the correlation between SFI and CMAP amplitude, which are hallmarks of axonal regeneration, was analyzed. As a result, a high correlation was found, suggesting a functional improvement in the experimental groups.

It appears encouraging that a single intraneural application of BoNT/A to the damaged nerve can be used to improve neural regeneration in peripheral nerves following a crush injury, and thus modulates regeneration and eventually improves functional outcomes. However, the blind method is difficult for the intraneural injection of BoNT/A in clinical practice. The sonography-guided method must be used, suggesting the need for an ultrasound device and an experienced clinician. In addition, when the perineurium is damaged by the needle, this treatment strategy may trigger secondary nerve damage. However, several studies have shown that the direct intranerve injection of BoNT/A induced no damage to nerves, which is in contrast with studies of other injectants, such as local anesthetics [45,46]. Perineural BoNT/A injections are not limited to animal models, and they have also been investigated in humans [47]. ESWT is non-invasive, and no special skills are required therefore, an extracorporeal shock wave can be applied more easily, with nerve regeneration outcomes comparable to BoNT/A under clinical settings.

This study has some limitations. Firstly, our results showed a difference between mRNA expression and protein levels in the time course of GAP43, ATF3, or NF200 after nerve injury. The moderate and varied correlations suggest that mRNA expression might sometimes be useful, but certainly far from perfect, in predicting protein expression levels [43]. Proteomic techniques might help improve our understanding of the relationship between mRNA expression and protein production. Secondly, although further investigations are needed to clarify the molecular mechanism underlying the effects of BoNT/A and ESWT in peripheral nerve injury, the data reported in this study demonstrate their positive effects on nerve regeneration. Thirdly, the numbers of myelinated nerve fibers and patterns of well-myelinated regenerating axons through morphological investigations were not determined in our study. Histological analysis will be needed in future experiments. Fourthly, further research is required to determine the type of ESWT and the number of sessions needed, as well as the requisite shock wave intensity. In addition, further researches are needed to establish the most effective BoNT/A injection method and the optimal dose for nerve regeneration. Considering that the perineural injection method is suitable for clinical use, additional research on perineural injection methodologies is needed in the future. These findings are particularly relevant in therapeutic approaches to normalize the disability caused by peripheral nerve injury.

## 4. Conclusions

Collectively, these findings indicate that BoNT/A and ESWT similarly induced the activation of Schwann cells with the axonal regeneration and functional improvement of damaged peripheral nerves. However, further research is needed to elucidate the molecular mechanism of these improvements. Although a significant volume of investigation is needed to clarify the mechanisms for neural regeneration, the research data in the present study should prompt further studies on the clinical application of BoNT/A and ESWT as a therapeutic strategy to promote nerve regeneration.

## 5. Materials and Methods

### 5.1. Animals and Surgical Procedure

Forty-five SPF Sprague Dawley (SD) rats were used in this study. Upon arrival, the rats were housed in three groups of standard cages (22 cm × 22 cm × 13 cm) at a constant temperature (23 ± 1 °C) under a 12-h light/dark cycle (06:30 a.m.–06:30 p.m.) with water and food ad libitum. They were approximately 6 weeks old and weighed 187–213 g at the time of surgery. Experiments were conducted from 08:30 a.m. to 12:30 p.m. Investigators were blinded to the subjects’ treatment. All procedures for animal experiments were performed in accordance with the principles outlined in the “Guide for the Care and Use of Laboratory Animals” (Institute for Laboratory Animal Research, Committee for the Update of the Guide for the Care and Use of Laboratory Animals, National Research Council of The National Academies, Washington, DC, USA; The National Academies Press: Washington, DC, USA, 2011) and approved by the “Institutional Animal Care and Use Committee” of Dongguk University (protocol code 201806176. Approval date: 6 July 2018).

Surgery was performed under 1–2% isoflurane anesthesia. An incision was made through the skin below the hip, and the muscle was blunt dissected using fine surgical scissors and forceps to expose the right sciatic nerve at midthigh level. Next, the sciatic nerve was crushed 1 cm above the region where it branched into sural, common peroneal, and tibial branches. The sciatic nerve was crushed using a Halsey needle holder (AE 064/13, NOPA, Tuttlingen, Germany) for 30 s [48]. The wound was then closed with VICRYL 3–0 (W9114, Ethicon LLC, Bridgewater Township, NJ, USA) and the rats were restored in a heated cage until all reflexes were normalized. Their sciatic nerves were harvested at 3, 6, or 10 weeks post-injury.

Sciatic nerve-crushed rats were divided into three groups: (1) rats (*n* = 15) injected with BoNT/A into the sciatic nerve through the crushed site immediately before suturing the wound; (2) rats (*n* = 15) applied with ESWT for the crushed sciatic nerve after suture; and (3) rats (*n* = 15, control) intraneurally injected with 0.9% saline (70 μL/kg) after sciatic nerve injury.

### 5.2. Experimental Groups

#### 5.2.1. Pharmacological Treatment

The nerve-crush site was intraneurally injected with onabotulinum toxin A (BoNT/A; Allergan, Irvine, CA, USA) using a 50-µL Hamilton Syringe (HAMILTON CO., NO706, Hamilton, OH, USA) immediately before suturing the wound. The dose of BoNT/A was based on previous studies demonstrating high efficacy at a low concentration with no side effects [18,28]. Botulinum toxin 100 U is equivalent to 4.8 ngtox [49]. Injectable solutions of BoNT/A (100 U) were freshly prepared via dilution in 1-mL normal saline (0.9% NaCl). A single session of intranerve BoNT type A (7 U/Kg; 336 pgtox/kg body weight) was used to compare the effect with ESWT in this study.

#### 5.2.2. Extracorporeal Shock Wave Therapy

After surgical suture, ESWT was applied to the crushed area. Ultrasound transmission gel (Aquasonic 100, Parker Laboratories Inc., Fairfield, NJ, USA) was used as the interface between the skin and shock wave area. Four hundred pulses of ESWT were applied at a dose of 0.098 mJ/mm^2^ and a frequency of 4 Hz in a single session using the REGENWAVE (HNT Medical, Seoul, Korea) with a 10-mm focus. This energy level was determined based on the results of the previous study [35].

### 5.3. Neurophysiology Test

The serial electrophysiological studies were performed every week for 10 weeks. The CMAP was recorded in deeply anesthetized animals (1–2% isoflurane). The sciatic nerve was stimulated percutaneously by single pulses of 0.3-ms duration delivered through a pair of bar electrodes placed at the sciatic notch. The CMAP of gastrocnemius medialis was recorded with microneedle electrodes. The reference electrode was attached to the Achilles tendon insertion site. All potentials were amplified and displayed on an electromyography machine (Synergy, Viasys Healthcare) at appropriate settings to measure the amplitude. The amplitudes of CMAP were recorded once supramaximal stimulation was reached by using progressively increasing stimulus intensity. [17]

### 5.4. Behavioral Tests

The progressive restoration of motor function was monitored by analyzing individual gait patterns and measuring multiple footstep parameters to calculate the Sciatic Functional Index (SFI). Footsteps were recorded by immersing the hindpaws of rat in black ink and allowing them to walk along a Perspex runway corridor (12 cm × 12 cm × 60 cm). Footstep parameters were calculated from a minimum of 5 footsteps recorded on 3 different tracks. Rats were tested every 7 days from weeks 1 to 10. SFI was evaluated using the equation [SFI = −38.3 × (EPL − NPL)/NPL + 109.5 × (ETS − NTS)/NTS + 13.3 × (EIT − NIT)/NIT − 8.8] [50]. Analysis of the footsteps involved measuring the length between the heel and third toe (PL: print length), the length between the first and fifth toes (TS: toe spread), and the length between the second and fourth toes (IT: intermediate toe spread). Experimental (E) and normal (N) data for both the right and left footsteps were recorded. The SFI of a normal rat is generally around 0, whereas that of a severely injured rat is close to −100.

### 5.5. Immunostaining of Sciatic Nerve

Rats were anaesthetized with 1–2% isoflurane (Sigma-Aldrich, St. Louis, MO, USA) at weeks 3, 6, and 10. The 10-mm segment of sciatic nerve distal to the crushed nerve site was removed. The entire tissue was placed in the base mold and covered with cryo-embedding media (OCT compound). The base mold containing the tissue block was placed in liquid nitrogen until the entire tissue block was submerged in liquid nitrogen to ensure complete freezing of the tissue. The frozen tissue block was stored at −80 °C until it was ready for sectioning.

Immunofluorescence staining was performed with 10-µm thick frozen tissue sections, which were then transferred onto glass slides. The tissue sections were dried at room temperature for 1 h and fixed with 4% paraformaldehyde solution (PFA; Sigma-Aldrich, Milan, Italy) at 4 °C for 1 h. After rinsing the fixed section in phosphate-buffered saline (PBS), the tissues used to apply GAP43 and ATF3 antibodies were permeabilized with 3% triton xX-100 solution before blocking. The fixed sections were rinsed in PBS and the slides were incubated in 5% BSA solution (Sigma-Aldrich, Milan, Italy) at room temperature for 1 h. Subsequently, the sections on the slides were treated with diluted primary antibodies and incubated in a humidified chamber overnight at 4 °C. Antibodies were purchased from Cell Signaling Technology, Inc. (Danvers, MA, USA). The effects of BoNT/A and ESWT on the Schwann cell activity of an injured sciatic nerve were evaluated by the expression of glial fibrillary acid protein (GFAP) and astroglial calcium-binding protein S100β (S100β), proteins expressed by nonmyelinating and myelinating Schwann cells, respectively [27,51]. Axonal regrowth was assessed by the expression of growth associated protein 43 (GAP43), activating transcription factor 3 (ATF3), and neurofilament 200 (NF200) [7,52,53]. The primary antibodies used were GFAP (1:200 dilution), GAP43 (1:250 dilution), NF200 (1:200 dilution), ATF3 (1:100 dilution), and S100β (1:200 dilution). The next day, the slides were rinsed in PBS and treated with a diluted secondary antibody (anti-mouse IgG and anti-Rabbit IgG2 at 1:200~1:500 dilution) and incubated in a humidified chamber at room temperature for one hour. After the slides were rinsed in PBS, the samples were coverslipped using a mounting solution containing DAPI and observed under a confocal microscope (STELLARIS, Leica Microsystem, Wetzlar, Germany).

### 5.6. ELISA

To measure the expression of proteins related to nerve regeneration in the collected frozen sciatic nerve tissue, the protein levels of GFAP, GAP43, S100β, NF200, and ATF3 were measured using an ELISA kit (Mybiosource, CA, USA) according to the manufacturer’s instructions.

### 5.7. Real-Time PCR

Real-time PCR was carried out to evaluate the expression of genes related to nerve regeneration in the collected frozen sciatic nerve tissue. The cryosectioned frozen tissue was placed in a 1.5-mL microtube, and the total RNA was isolated using an RNase mini prep kit (Qiagen Inc., Germantown, MD, USA). Thereafter, the total RNA was quantified using a Nanodrop spectrophotometer, and cDNA was synthesized using 1 µg of total RNA (Intron Inc. LTD, Seoul, Korea). Real-time PCR was performed using LightCycler 480 SYBR green I master (Roche Inc., Branchburg, NJ, USA). Gene primers were GFAP, GAP43, NF200, ATF3, and S100β, and GAPDH was used as an internal control. The primer sequences and product size are shown in Table 1.

### 5.8. Western Blot

Western blot was performed to measure the expression of nerve regeneration-related proteins in the collected frozen sciatic nerve tissue. The cryosectioned frozen tissue was placed in a 1.5-mL microtube, to which RIPA buffer-containing protease-phosphatase was added and incubated on ice for 30 min to lyse the tissue. Next, the total protein was quantified using the BCA method, and electrophoresis was performed using 10–12% sodium dodecyl sulfate (SDS) polyacrylamide gel. After blotting the protein with PVDF membrane, blocking was performed for 1 h at room temperature using 5% BSA solution. Membranes were incubated overnight at 4 °C with the following primary antibodies: GFAP rabbit mAb (1:1000 dilution, Cell Signaling Technology, Inc., Danvers, MA, USA), GAP43 rabbit mAb (1:1000 dilution, Cell Signaling Technology, Inc., Danvers, MA, USA), NF200 rabbit pAb (1:1000 dilution, Sigma Aldrich, Port Washington, WI, USA), ATF3 rabbit mAb (1:1000 dilution, Cell Signaling Technology, Inc., Danvers, MA, USA), and S100β mouse mAb (1:2000 dilution, AbCam, Inc., Eugene, OR, USA). After a reaction with the primary antibodies, the samples were washed 4 times for 3 min each with TBST, followed by incubation with a secondary antibody (anti-mouse IgG and anti-Rabbit IgG2 at 1:2000~1:4000 dilution, Cell Signaling Technology, Inc., Danvers, MA, USA) solution for 1 h at room temperature. After a reaction with the ECL solution (Thermo Fisher Scientific, Eugene, OR, USA), it was developed using an LAS 3000 imaging system. Beta actin (1:1000 dilution, Cell Signaling Technology, Inc., Danvers, MA, USA) was used as an internal control.

### 5.9. Statistical Analysis

The results were expressed as the mean values ± standard error of the mean or standard deviation in graphic and text representations. The differences between the 3 groups at each time point were evaluated with one-way ANOVA followed by Tukey’s multiple comparison test using GraphPad Prism version 9 for Windows (GraphPad Software; San Diego, CA, USA). The correlation between the SFI score and CMAP amplitude was assessed using a Pearson correlation analysis. Statistical significance was set at *p* < 0.05.

## Figures and Tables

**Table 1:** Primer sequences.

Genes	Forward Primer	Reverse Primer
GFAP	5′-AGT GGT ATC GGT CCA AGT TTG C-3′	5′-TGG CGG CGA TAG TCA TTA GC-3′
S100β	5′-GCC CTC ATT GAT GTC TTC C-3′	5′-TCC TTT AGT TTC TCG TCC TTC-3′
GAP43	5′-AAG AAG GAG GGA GAT GGC TCT-3′	5′-GAG GAC GGC GAG TTA TCA GTG-3′
ATF3	5′-CCT GCA GAA GGA GTC AGA GAA-3′	5′-CGT TCT GAG CCC GGA CGA TA-3′
NF200	5′-GGA GGA GAG CCG TCA GGT AGA C-3′	5′-TTT CTG TAA TCA GCA GCG ATC TCA AT-3′
GAPDH	5′-GGC ACA GTC AAG GCT GAG AAT G-3′	5′-ATG GTG AAG ACG CCA GTA-3′

## Data Availability

Data are contained within the article.

## Abbreviations

ATF3	Activating transcription factor 3
BoNT/A	Botulinum neurotoxin A
CI	Confidence intervals
CMAP	Compound muscle action potential
ESWT	Extracorporeal shock wave therapy
*GAP43*	Growth associated protein 43
*GFAP*	Glial fibrillary acid protein
MD	Mean difference
NF200	Neurofilament 200
SMD	Standardized mean difference
